# Pericardial Effusion and Tamponade Complicating Acute Pericarditis in a Human Metapneumovirus-Infected Adult: A Unique Case Report

**DOI:** 10.7759/cureus.8454

**Published:** 2020-06-05

**Authors:** Nawfal Mihyawi, Ayman R Fath, Dawood Findakly, Arnold Forlemu, Raina R Roy

**Affiliations:** 1 Internal Medicine, Creighton University Arizona Health Education Alliance/valleywise Health Medical Center, Phoenix, USA; 2 Internal Medicine, Creighton University Arizona Health Education Alliance/Valleywise Health Medical Center, Phoenix, USA; 3 Cardiology, Creighton University Arizona Health Education Alliance/Valleywise Health Medical Center, Phoenix, USA

**Keywords:** acute pericarditis, human metapneumovirus, leukocytosis, pericardial effusion, cardiac tamponade

## Abstract

Acute pericarditis (AP), or pericardial sac inflammation, is a self-limited condition in healthy individuals. Viruses, including adenoviruses, enteroviruses, cytomegalovirus, and influenza virus, have been well documented to cause AP. In contrast, human metapneumovirus (hMPV), a relatively newer virus, has been described in a few cases to cause serious cardiac complications. Here we report a patient who developed an imminent cardiac tamponade associated with hMVP respiratory infection.

## Introduction

Human metapneumovirus (hMPV) is a relatively newer virus that was first diagnosed in 2001. It belongs to the Pneumoviridae family, which is a group of large enveloped negative-sense single-stranded RNA viruses [1]. The hMPV causes respiratory tract infections (RTIs), mainly in children, and to a lesser degree in adults. It is usually associated with mild, self-limited upper, or lower RTIs in immunocompetent adults [2]. In this report, we present a case of a young, healthy male who developed large pericardial effusion associated with hMPV infection.

## Case presentation

**Figure 1:**
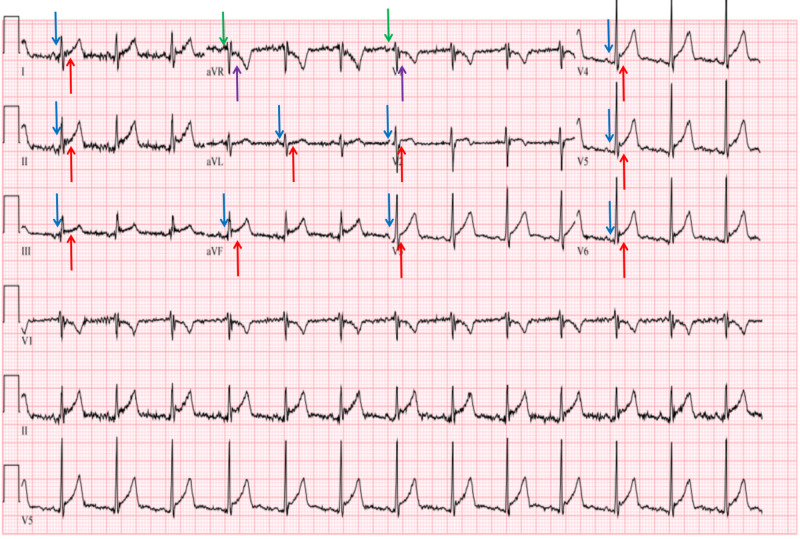
Acute pericarditis electrocardiographic findings of diffuse ST elevations (red arrows) and PR depression (blue arrows) in all leads except aVR and V1, which show reciprocal ST depression (purple arrows) and PR elevation (green arrows).

A 26-year-old healthy man diagnosed clinically with uncomplicated idiopathic acute pericarditis (AP). He was treated appropriately with ibuprofen and colchicine where symptoms of chest pain and shortness of breath were significantly improved on an outpatient follow-up visit (Figure 1). One month later, he presented to the emergency department with three days of progressive chest pain, fever, dry cough, and difficulty breathing. The chest pain was sharp, at the left side of the chest, radiated to the left shoulder, worse when supine and improved with sitting up. The patient reported that he had been in close contact with his four-year-old son, who had a recent upper RTI. At the time of presentation, the patient was tachycardic with a heart rate of 115 bpm, mildly hypertensive with a blood pressure of 145/85 mmHg, afebrile, and had a normal respiratory rate. Physical exam was notable only for distant heart sounds on chest auscultation, with normal neck veins and negative Kussmaul's sign. Laboratory studies were pertinent for peripheral white blood cells of 11.0 x 10^3/µL with elevated absolute neutrophil count and absolute monocyte count at 7.4 x 10^3/µL and 1.2 x 10^3/µL, respectively, and with normal absolute lymphocyte count and absolute eosinophil count at 1.5 x 10^3/µL and 0 x 10^3/µL, respectively. His C-reactive protein was 92 mg/L, erythrocyte sedimentation rate was 33 mm/hr, D-Dimer was 359 ng/ml, and troponin was <0.012 ng/ml.

**Figure 2:**
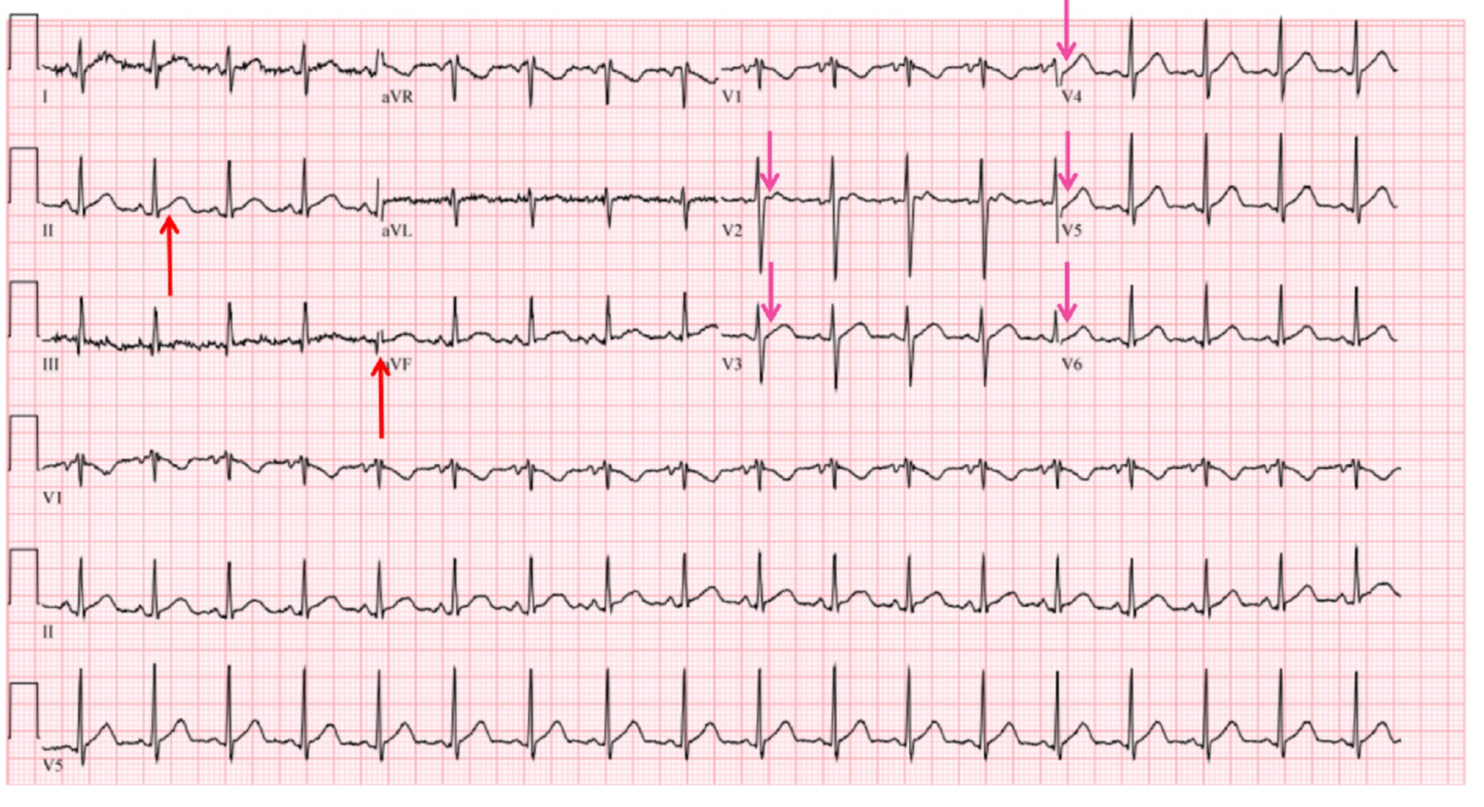
When compared with the prior electrocardiogram, ST segments are less elevated in the inferior leads (red arrows) and no longer elevated in the anterior leads (pink arrows).

A repeat electrocardiogram (EKG) this time showed ST segments are less elevated in the inferior leads and no longer elevated in the anterior leads when compared to the initial EKG upon his first presentation one month earlier (Figure 2). 

**Figure 3:**
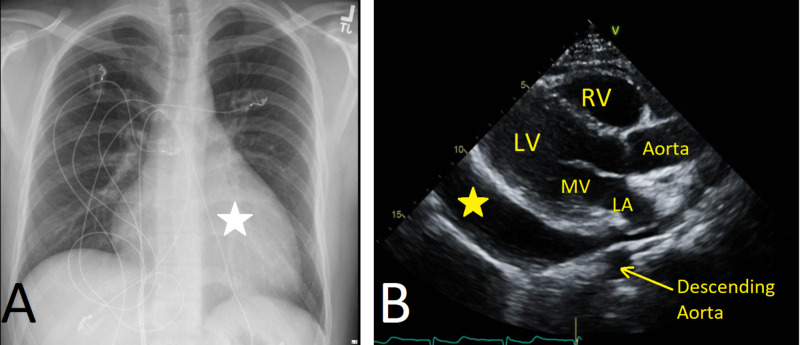
Imaging performed at the time of presentation. (A) Chest X-ray showing enlargement of the cardiac silhouette (white star). (B) Transthoracic echocardiography: parasternal long axis view showing pericardial effusion (yellow star). LV: left ventricle; MV: mitral valve; LA: left atrium; RV: right ventricle.

Rheumatological workup was negative for rheumatoid factor, antinuclear antibodies (ANA), and anti-double-stranded DNA (anti-dsDNA). Both HIV and hepatitis panel were negative. Respiratory viral pathogen panel was positive for hMPV by nasopharyngeal and oropharyngeal polymerase chain reaction (PCR), and negative for adenovirus, human rhinovirus/enterovirus, influenza A and B, human parainfluenza virus types 1-4, and respiratory syncytial virus (RSV). Chest X-ray revealed a significantly enlarged cardiac silhouette compared to baseline reading (Figure 3A). Echocardiography showed normal left ventricle contractility with an ejection fraction of 69%, large pericardial effusion, diastolic right ventricular collapse, exaggerated ventricular interdependence, and dilated inferior vena cava with reduced collapse, all consistent with echocardiographic features of cardiac tamponade (Figure 3B). 

**Figure 4:**
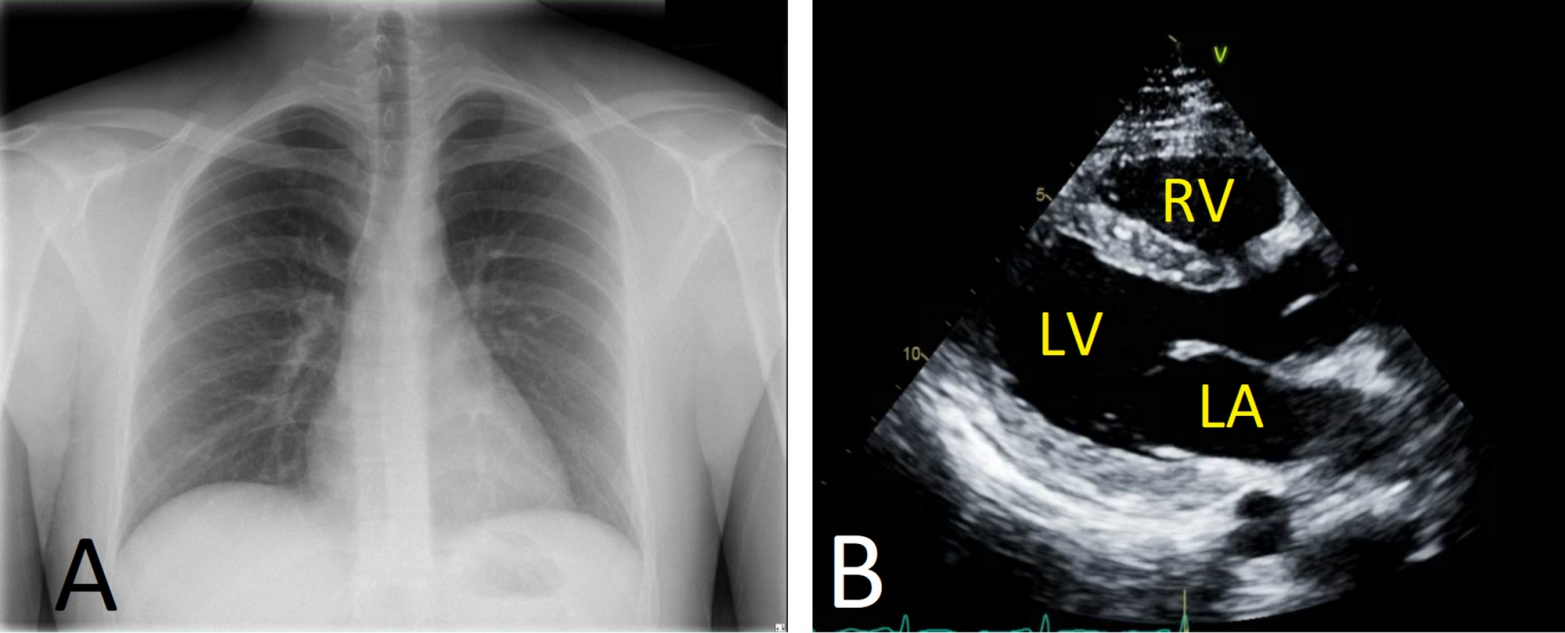
Imaging upon follow-up post pericardiocentesis. (A) Chest X-ray showing the normal size of the heart. (B) Transthoracic echocardiography: parasternal long axis view demonstrating resolution of pericardial effusion. LV: left ventricle; LA: left atrium; RV: right ventricle.

Hence, a diagnosis of hMPV-induced AP complicated with pericardial effusion and tamponade was made, and the patient was admitted to the intensive care unit (ICU) for close hemodynamic monitoring and further management. In the ICU, the patient was promptly started on prednisone and colchicine and underwent pericardiocentesis with approximately 800 ml of dark red blood was drained. The pericardial fluid culture was negative for fungi, bacteria, and Mycobacterium. The patient was discharged home on colchicine 0.6 mg bid for three months, and taper steroids. Follow-up showed improvement in symptoms with complete resolution of pericardial effusion based on the chest X-ray performed after the procedure and echocardiogram performed seven days after the first study (Figure 4). 

## Discussion

AP is characterized by the inflammation of the pericardial sac surrounding the heart [3]. It can be complicated by excessive pericardial fluid accumulation leading to pericardial effusion, which may eventually impede diastolic filling, with resultant hemodynamic compromise from cardiac tamponade physiology. Viruses such as adenoviruses, enteroviruses, cytomegalovirus, influenza virus, hepatitis B virus, and herpes simplex virus are well-known causes of AP [4]. In healthy individuals, infection by these viruses is often self-limited and follows a benign course and, therefore, further invasive interventions like pericardiocentesis are generally not needed [2]. hMPV is a new ribonucleic acid (RNA) that commonly causes a mild upper or lower RTI in all ages, with a higher prevalence in children [1,2]. It has been identified in 4%-16% of patients with RTI, where it often transmits by large aerosols and droplets of infected patients. Moreover, a nosocomial transmission has been reported in hospitalized patients and in skilled nursing facilities [5,6].

The exact incubation period of the virus is unknown, but it is thought to be between five and nine days [7]. The virus has a seasonal variation with a peak during late winter and early spring. Reverse-transcriptase PCR, or RT-PCR, is the most common and sensitive method used to diagnose hMPV when patients present with the most common hMPV manifestations of cough, rhinorrhea, nasal congestion, fever, hoarseness, dyspnea, and wheezing [1,8]. Although hMPV is typically a respiratory pathogen, a few case reports have described hMPV affecting the heart. The dilemma of whether hMPV has an increased propensity to infect the perimyocardium or patients with underlying cardiovascular disease are more vulnerable to hMPV has not been elucidated. In this regard, Zeng et al. suggested an increased seroprevalence of hMPV in elderly hypertensive patients living in long-term care facilities [9]. 

The pathogenesis of hMPV on the pericardium is unknown; however, like other cardiotropic viruses, hMPV may have direct pericardial cytotoxic or cytolytic effects. Moreover, it may lead to sustained inflammation and immune response to the pericardium through molecular viral proteins mimicry to cardiac proteins [10]. Our patient presented with AP that was associated with hMPV infection complicated by cardiac tamponade, requiring ICU admission and pericardiocentesis. He was discharged on steroids and colchicine and had a complete resolution of symptoms during outpatient follow-up. In our case, hMPV was not isolated from the pericardial fluid, and it was presumed to be secondary to hMPV infection, given the correlation of symptoms with virus detection in the respiratory swab, and all other workup was negative. Moreover, if hMPV infection leads to cardiac complications via molecular mimicry of its proteins to those of the heart, detecting in pericardial fluid analysis or biopsy may not be needed to definitively tie hMPV as a cause of pericarditis.

Few cases reported hMPV affecting the myocardium [11,12]. This patient was diagnosed with hMPV infection, complicated by AP. To the best of our knowledge, only one reported case of a healthy 62-year-old woman who developed AP secondary to hMPV was described in the literature, therefore making our patient the second published case that addresses the association between hMPV and AP [13].

## Conclusions

This unique association between hMPV and AP should be part of the differential diagnosis in healthy adults with recent upper RTI leading to progressively worsening chest pain. Further studies are required to determine the true incidence of hMPV-induced AP, and the implied risk of life-threatening cardiovascular complications, including cardiac tamponade, in order to facilitate early intervention, which will further improve outcomes.
